# The use of chemical markers for the identification of farm escapees in feral mink populations

**DOI:** 10.1007/s10646-014-1213-y

**Published:** 2014-02-27

**Authors:** Marcin Brzeziński, Andrzej Zalewski, Agnieszka Niemczynowicz, Ingeborga Jarzyna, Małgorzata Suska-Malawska

**Affiliations:** 1 Faculty of Biology, Biological and Chemical Research Centre, University of Warsaw, ul. Żwirki i Wigury 101, 02-089 Warsaw, Poland; 2 Mammal Research Institute, Polish Academy of Sciences, 17-230 Białowieża, Poland

**Keywords:** *Neovison vison*, Contamination, Chemical markers, Mercury

## Abstract

Variations in the contaminant burden in feral and ranch mink, resulting from differences in their diet, may permit the identification of farm escapees. However, this is only possible in the case of contaminants that accumulate to significantly different levels in the two groups of animals. The main objective of this study was to identify chemical markers whose concentrations differ between feral and ranch mink, by analyzing the accumulation of 13 chemical elements in liver and kidney samples. Total mercury levels were up to 15-fold higher in kidney, and up to 7-fold higher in liver of feral mink compared with ranch mink. The majority of feral mink samples analyzed for mercury, contained concentrations that ranged from 1 to 5 μg/g in kidney (68 %) and from 1 to 5 μg/g in liver (70 %). In comparison, the organs of ranch mink had significantly lower levels of mercury: 95 % of kidney samples had concentrations below 1 μg/g and 82 % of liver samples had concentrations below 1 μg/g. Small geographical variations in Hg levels were observed in mink from the four studied feral populations. Significant differences in Cu concentrations between ranch and feral mink were also detected, with low variation within the two groups. Less pronounced differences were recorded for other chemical elements. These data suggest that Hg and Cu may be used as chemical markers for the identification of first generation mink farm escapees.

## Introduction

Wild-living American mink (*Neovison vison*) are considered to be good indicators of environmental contamination (Basu et al. 2007), although some authors have questioned whether mink is a reliable sentinel species (Bowman and Schulte-Hostedde 2009). Several arguments have been made against this notion, including the fact that many wild-living mink fail to meet the criterion of continuous residence, since they are escapees from farms. Bowman and Schulte-Hostedde (2009) rightly argued that to be a sentinel species, the source of the examined pollutant must be the environment in which the mink lives. In Poland, all feral mink populations are comprised of the distant descendents of ranch animals, but the inflow of first generation farm escapees may still be significant in some cases (Zalewski et al. 2010, 2011). Wherever mink are farmed, they escape; thus, the probability of admixture of ranch mink with the wild-living population increases in areas with a high density of mink farms. Bowman et al. (2012) recently demonstrated that in Canada contamination levels differ between free-ranging domestic mink and wild (native) mink, and concluded that due to this fact, ranch mink escapees and descendents, which supply feral populations, may bias studies of environmental contamination in which mink is treated as a sentinel species. In responding to the criticism of Bowman and Schulte-Hostedde (2009) on the use of mink as sentinel species, Basu et al. (2009) argued that despite escaping ranch mink supplementing free-ranging populations, often it will be only the most recent arrivals that fail to reflect local conditions. In other cases, artificial selection may lead to different foraging behaviours in escaped domestic mink compared to wild mink. Due to the rapid assimilation of most contaminants through the diet, escapees that survive in the wild for a prolonged period, and more so their descendents, will bioaccumulate pollutants present in the environment. Unfortunately, the results of genetic studies that confirm the inflow of ranch mink to feral populations cannot show precisely how long particular escapees have survived in the wild (Basu et al. 2009).

Significant variation in the contaminant burdens in feral and ranch mink, resulting from differences in the diets of these animals, may permit the identification of first generation ranch escapees that have only joined a feral population relatively recently. However, due to the expected variation in pollutant accumulation in feral mink, estimation of the proportion of mink of recent farm origin in feral populations based on contamination burdens may be possible only for particular contaminants that show significantly different levels of accumulation in the two groups of animals.

American mink is a carnivore at the top of a food chain and so bioaccumulates pollutants that are present in the tissues of consumed prey. In the wild, the mink diet varies greatly according to the habitat and season, but aquatic and semi-aquatic prey, such as crayfish, fish and amphibians, usually predominate (Jędrzejewska et al. 2001; Bartoszewicz and Zalewski 2003; Brzeziński 2008). The diet composition of ranch mink differs from that of feral mink: on farms the mink are fed mainly fish and chicken fodder (Gliński and Kostro 2002). Differences in the nutrition of feral and ranch mink should result in different concentrations of pollutants, including heavy metals, in their tissues. Moreover, as all ranch mink from a certain farm consume the same food, rather low variability of contamination among ranch individuals may be expected. In contrast, the variation in the levels of pollutants in the tissues of feral mink individuals should be higher, since these animals often hunt a wide variety of different prey (Sidorovich 2000; Brzeziński 2008).

The estimation of the inflow of ranch mink escapees to existing feral populations is an important goal of conservation biology and any method with the potential to distinguish between ranch and feral mink, should be tested. We hypothesize that first generation mink escapees, fed on specific fodder before their escape, should have significantly different concentrations of some chemical elements in their tissues, compared with feral mink. Differences in element concentrations in tissue samples from ranch and feral mink would allow these two groups to be distinguished using a quantitative measure.

To assess the utility of element concentrations (major, minor and trace) as a measure of long-term dietary accumulation in ranch and feral mink we (i) examined spatial variation in the concentrations of 13 elements (Cu, Zn, Hg, Pb, Se, Cr, Mo, Mn, Ni, Sr, Tl, Co, Cd) in the liver and kidney of feral mink trapped in four national parks in northern Poland, (ii) compared the mean levels and ranges of these elements in the tissues of feral and ranch mink to determine whether the concentrations of any particular elements significantly differentiate these two groups, and (iii) compared element concentrations across age groups and between the sexes in the mink.

## Materials and methods

### Sample collection

**Fig. 1:**
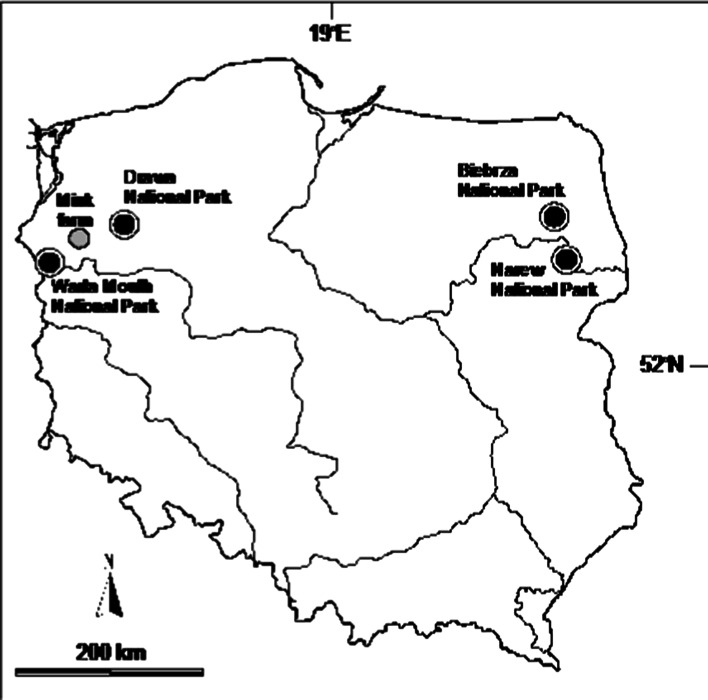
Location of four national parks and mink farm

Concentrations of chemical elements were measured in the kidney and liver of 111 feral mink trapped in four national parks and in 22 ranch mink from one farm. Two of the national parks, Biebrza NP (BNP; 26 mink) and Narew NP (NNP; 29 mink), were situated in northeastern Poland, whereas the other two, Warta Mouth NP (WMNP; 30 mink) and Drawa NP (DNP; 26 mink), as well as the mink farm, were in northwestern Poland (Fig. 1).

In the national parks, feral mink carcasses were collected during mink eradication programmes. Mink were trapped along the main river (names of national parks correspond to the river names) using live wire-mesh traps (18 × 15 × 65 cm) baited with fresh fish, deployed on the river banks. Mink trapping was conducted in a series of five trapping sessions (March–April and November–December in the years 2009–2011) by staff of the national parks. The mink carcasses were sexed and dissected. Livers and kidneys were removed and stored at −20 °C prior to chemical analysis. Subadults (<1 year old) and adults were distinguished by measurement of the ratio of pulp cavity to tooth width from X-rays of the canines. Mink with a pulp to width ratio of over 0.3 were assumed to be subadults (Helldin 1997).

### Analytical procedures

Chemical analyses were conducted in the Laboratory of Environmental Chemistry at the Biological and Chemical Research Centre, University of Warsaw, Poland. The total concentration of 13 chemical elements (Cu, Zn, Hg, Pb, Se, Cr, Mo, Mn, Ni, Sr, Tl, Co, Cd) were quantified in liver and kidney samples using an ultrasound (US)-assisted extraction procedure for the estimation of element concentrations (Santos et al. 2006). Parameters affecting extraction efficiency, including extract type and concentration, sonication time and amplitude, were optimized to obtain quantitative recoveries of all elements. A procedure using 1 % (v/v) HNO_3_ was rapid (15 min) and accurate for most of the elements. Element solubilization from tissue samples of 100 mg was achieved by 4 min of sonication at an amplitude of 40 %. The overall precision was over 10 % (Welna et al. 2011). The sample extracts were analyzed using inductively coupled plasma mass spectrometry (ICP-MS, Perkin Elmer ELAN 6100 DRC). Chemical analysis of the total Hg concentration was conducted using an advanced mercury analyser (AMA254 LECO Mercury Analyzer, USA) equipped with an autosampler. The analytical procedures were quantified using certified reference materials (CRM 185 R). CRM were used to perform a standard material test to ensure the precision and accuracy of the analysis with element recovery rates for the CRM of between 91 and 109 % for samples. The calibration curves for all the studied elements covered the concentration range 0.01–1.0 mg/dm^3^. Each sample was analyzed in duplicate and results were accepted only if the variance between duplicates did not exceed that CRM standard.

Of the 13 chemical elements analyzed in mink kidney, Sr and Tl were detected in less than 10 % of the samples, and Co, Mo and Cd in less than 50 % (Table 1). In liver samples, Co, Cd and Tl were detected in less than 10 %, and Sr and Ni in less than 50 %. As the concentrations of Tl, Sr, Co and Cd were below the detection limits in most kidney and liver samples, these four elements were omitted from further analyses.

**Table 1:** Detection limits (μg/g dry weight) of the individual elements and the proportion of kidney and liver samples in which element concentrations were below these limits

Element	Detection limit	% of samples	
		Kidney	Liver
Cu	0.10	9.8	0
Sr	1.72	**97.8**	**69.2**
Zn	9.69	0	0
Cd	0.30	**60.9**	**96.2**
Hg	0.26	8.3	4.5
Tl	0.35	**99.2**	**99.2**
Pb	0.23	33.1	27.8
Se	0.59	0.8	0
Cr	0.12	0	44.4
Mo	0.89	60.9	18.8
Mn	0.41	0	0
Co	0.06	**50.4**	**91.7**
Ni	0.04	13.5	65.4

### Materials and reagents

All reagents were of analytical grade. The calibration standards were prepared by diluting the stock multi-elemental standard solution in 0.5 % (v/v) nitric acid. A commercial ICP multi-element standard solution IV Merck Millipore (23 elements in diluted nitric acid) 1,000 mg/l: Ag, Al, B, Ba, Bi, Ca, Cd, Co, Cr, Cu, Fe, Ga, In, K, Li, Mg, Mn, Na, Ni, Pb, Sr, Tl, Zn Certipur^®^ and separately a Hg standard for Advanced Mercury Analyzer AMA254 were used. As certified reference materials we used bovine liver BCR^®^-185R (Sigma Aldrich^®^). Ultrapure MilliQ water (Millipore, France) was used for dilutions. High purity supplier argon was employed as the inert carrier gas.

### Data analysis

Contaminants in feral and ranch mink tissues were examined by pooling according to spatial variations (geographical factors), regardless of demographic factors (sex, age, body mass). A one-way analysis of variance (ANOVA) with multiple comparisons, implemented using Statistica for Windows, was used to determine whether major, minor and trace element levels differed between locations and feral and ranch populations. Differences were considered significant when *P* < 0.05. Tukey’s post hoc tests on means were used to examine differences between the tissues of feral mink from various national parks and those of ranch mink. If data failed the assumption of equal variance (Levene’s Test), comparisons were made using Kruskal–Wallis one way analysis of variance by rank; post hoc testing was conducted using non-parametric multiple comparisons for unequal sample sizes (Zar 1998). Spearman rank correlation analyses were performed to examine the relationships between element concentrations and spatial and demographic variations. Moreover, feral mink-ranch mink relationships for the mercury and copper concentrations in kidney and liver were determined by means of principal components analysis (PCA) using Canoco for Windows Version 4.0 (ter Braak and Šmilauer 1998).

## Results

In both groups of mink, the mean concentrations of Cu, Se, Cr, Mo and Mn differed significantly (*P* < 0.0001) in kidney and liver samples. Levels of Cu were about 5-fold higher in liver compared to kidney in ranch mink, and about 1.5-fold higher in feral mink. Similarly, Mo and Mn concentrations were 1.5- to 2-fold higher in liver than in kidney in both groups of mink. Lower mean concentrations in liver compared to kidney were recorded for Se and Cr in all mink.

Only a few significant correlations between various element concentrations in mink tissues were identified (Spearman rank correlation). Selenium concentrations in ranch mink kidneys were positively correlated with those of chromium (r^2^ = 0.47, *P* < 0.05), manganese (r^2^ = 0.85, *P* < 0.05) and zinc (r^2^ = 0.84, *P* < 0.05). In feral mink kidneys, positive correlations were found between the concentrations of selenium and copper (r^2^ = 0.54, *P* < 0.05). Levels of selenium and manganese (r^2^ = 0.65, *P* < 0.05), and zinc and manganese (r^2^ = 0.64, *P* < 0.05) correlated positively in the livers of ranch mink.

In general, all investigated mink showed little variation in the mean levels of the examined elements, but some differences were recorded. Significant variation was observed in the nephric concentrations of copper (F_4,128_ = 4.74, *P* = 0.001), zinc (F_4,128_ = 6.74, *P* < 0.0001) and mercury (F_4,128_ = 14.46, *P* < 0.0001), and the hepatic concentrations of selenium (F_4,128_ = 5.64, *P* = 0.003), zinc (F_4,128_ = 8.41, *P* < 0.0001) and mercury (F_4,128_ = 9.19, *P* < 0.0001) (Tables 2, 3).

**Table 2:** Concentration of chemical elements (μg/g dry weight) in kidney of ranch and feral mink from Poland collected in 2009–2011

Element	Ranch mink	Feral mink				Statistics
		DNP^a^	WMNP^b^	BNP^c^	NNP^d^	
	*n* = 22	*n* = 26	*n* = 30	*n* = 29	*n* = 26	
Cu	**8.64 (7.26)** ^**acd**^	17.80 (5.78)	15.00 (13.85)	16.99 (4.65)	16.87 (4.76)	F_4,128_ = 4.74, *P* = 0.001
Range:	nd–21.73	nd–26.29	nd–65.14	5.88–27.16	5.93–29.49	
Zn	**53.67 (35.36)** ^**ac**^	92.92 (63.42)	**70.23 (22.41)** ^**c**^	**115.40 (67.01)** ^**d**^	76.22 (15.46)	F_4,128_ = 6.74, *P* < 0.0001
Range:	15.28–193.65	50.11–344.60	30.70–126.99	46.61–360.58	28.72–106.47	
Hg	**0.28 (0.33)** ^**abcd**^	4.68 (2.66)	3.50 (2.19)	4.39 (3.44)	4.11 (1.19)	F_4,128_ = 14.46, *P* < 0.0001
Range:	nd–1.18	1.34–12.80	0.81–9.82	nd–13.72	2.51–8.14	
Pb	0.47 (0.80)	0.88 (1.19)	0.75 (1.13)	0.95 (0.73)	0.89 (0.93)	H = 10.96, *P* = 0.02
Range:	nd–2.33	nd–4.65	nd–4.25	nd–2.34	nd–4.34	
Se	4.55 (1.22)	5.17 (0.77)	4.80 (1.43)	4.44 (1.27)	5.25 (1.29)	H = 9.09, *P* = 0.05
Range:	1.60–6.20	3.40–6.43	0.87–6.65	nd–6.67	2.42–9.24	
Cr	0.65 (0.31)	0.62 (0.34)	0.71 (0.57)	0.49 (0.34)	0.79 (0.47)	H = 12.59, *P* = 0.01
Range:	0.21–1.52	0.25–1.65	0.21–3.40	0.26–2.04	0.26–2.39	
Mo	0.53 (0.77)	0.60 (1.24)	0.87 (1.46)	0.47 (0.83)	1.10 (1.49)	Not significant
Range:	nd–3.21	nd–5.06	nd–6.59	nd–3.27	nd–4.64	
Mn	2.83 (1.33)	2.78 (0.54)	2.90 (0.95)	2.98 (0.71)	3.34 (0.64)	H = 12.19, *P* = 0.01
Range:	1.06–7.69	1.54–3.88	0.89–4.78	1.17–4.85	2.03–4.47	
Ni	0.57 (1.28)	0.29 (0.25)	0.66 (1.06)	0.91 (1.41)	1.17 (1.53)	H = 16.74, *P* = 0.002
Range:	nd–5.76	nd–0.77	nd–4.72	nd–4.97	nd–7.95	

Values represent the mean, (SD) and range, n denotes the number of mink analyzed; *nd* not detected (below detection limit). Statistics denote the results of one-way parametric and non-parametric analysis of variance tests (F and H statistics, respectively) with associated *P* values

Different letters indicate significant differences in element concentrations among sites. Significant differences are denoted by superscript (bold) letters (Tukey’s post hoc test)

*DNP* Drawa National Park, *WMNP* Warta Mouth NP, *BNP* Biebrza NP, *NNP* Narew NP

**Table 3:** Concentration of chemical elements (μg/g dry weight) in liver of ranch and feral mink from Poland collected in 2009–2011

Element	Ranch mink	Feral mink				Statistics
		DNP^a^	WMNP^b^	BNP^c^	NNP^d^	
	*n* = 22	*n* = 26	*n* = 30	*n* = 29	*n* = 26	
Cu	**42.81 (22.27)** ^**abcd**^	28.06 (9.37)	28.05 (17.16)	27.95 (11.11)	22.38 (10.33)	F_4,128_ = 6.51, *P* < 0.00001
Range	14.25–124.98	13.63–55.01	9.64–94.10	10.62–61.22	11.67–65.62	
Zn	**71.62 (31.69)** ^**c**^	**112.22 (79.50)** ^**b**^	67.22 (21.99	**135.38 (71.13)** ^**d**^	82.73 (26.71)	F_4,128_ = 8.41, *P* < 0.00001
Range	30.48–146.58	39.15–402.49	39.52–125.38	57.91–402.35	46.25–159.67	
Hg	**0.61 (0.72)** ^**abcd**^	**5.04 (3.54)** ^**b**^	2.60 (1.89)	4.78 (3.44)	4.22 (3.84)	F_4,128_ = 9.19, *P* < 0.00001
Range	nd–2.62	0.84–13.39	0.55–9.89	0.51–12.13	1.48–17.75	
Pb	0.79 (0.92)	**1.20 (1.51)** ^**b**^	0.25 (0.43)	1.05 (0.83)	0.66 (0.34)	H = 25.81, *P* < 0.00001
Range	nd–3.09	nd–7.24	nd–1.75	nd–3.35	nd–1.46	
Se	**1.67 (0.75)** ^**a**^	**3.11 (2.03)** ^**bc**^	1.90 (0.46)	2.02 (0.77)	2.58 (1.46)	F_4,128_ = 5.64, *P* = 0.0003
Range	0.87–4.39	0.99–9.29	0.97–2.82	0.94–3.47	1.37–8.43	
Cr	**0.30 (0.37)** ^**b**^	**0.25 (90.50)** ^**b**^	0.10 (0.19)	0.34 (0.41)	0.48 (0.67)	F_4,128_ = 2.69, *P* = 0.03
Range	nd–1.31	nd–2.34	nd–0.60	nd–1.68	nd–3.17	
Mo	1.03 (1.25)	1.42 (1.25)	1.16 (1.01)	1.77 (1.49)	1.74 (1.19)	H = 15.99, *P* = 0.003
Range	nd–4.66	nd–5.90	nd–4.72	nd–7.99	nd–6.78	
Mn	**5.02 (3.26)** ^**ac**^	7.54 (2.68)	6.87 (1.87)	7.64 (3.77)	7.13 (1.27)	F_4,128_ = 3.68, *P* = 0.007
Range	2.26–17.22	4.14–18.21	3.26–12.58	4.36–23.22	4.70–10.10	
Ni	0.18 (0.35)	0.27 (0.52)	0.04 (0.12)	0.15 (0.27)	0.16 (0.43)	H = 11.47, *P* = 0.02
Range	nd–1.39	nd–2.17	nd–0.52	nd–1.06	nd–2.25	

Values represent the mean, (SD) and range, *n* denotes the number of mink analyzed; *nd* not detected. Statistics denote the results of one-way parametric and non-parametric analysis of variance tests (F and H statistics, respectively) with associated *P* values

Different letters indicate significant differences in element concentrations among sites. Significant differences are denoted by superscript (bold) letters (Tukey’s post hoc test)

*DNP* Drawa National Park, *WMNP* Warta Mouth NP, *BNP* Biebrza NP, *NNP* Narew NP

### Effect of sex, body mass and age on nephric and hepatic concentrations of elements

There was no significant sex effect on the mean concentrations of the majority of chemical elements in the kidneys of both ranch and feral mink. However, significantly higher mean nephric copper concentrations were recorded in feral females compared to feral males (H = 3.88, *P* = 0.04), and the opposite sex effect was recorded for molybdenum (H = 7.65, *P* = 0.005). In liver, the only element whose concentrations differed between the sexes in feral mink was copper (H = 9.05, *P* = 0.002), and similarly to kidney, higher concentrations were recorded in females than in males.

An effect of body mass on element concentrations within sex groups of ranch and feral mink was found for only a few elements. A positive correlation between body mass and the nephric concentration of chromium was recorded in ranch mink males (r^2^ = 0.66, *P* < 0.05), while a negative correlation was detected with the concentration of zinc in feral males (r^2^ = −0.35, *P* < 0.05). In liver, differences in element burdens related to body mass were recorded for ranch females: larger individuals had higher concentrations of manganese (r^2^ = 0.63, *P* < 0.05). In both feral males and females, body mass was negatively correlated with the hepatic concentration of zinc (r^2^ = −0.41, *P* < 0.05; r^2^ = −0.40, *P* < 0.05, respectively).

Age effects were not observed for the concentrations of any chemical element in the kidneys or livers of feral mink, except for selenium in the former. Adult animals had significantly higher nephric levels of Se than mink under 1 year old (H = 4.67, *P* = 0.003).

### Differences between the kidney and liver element concentrations of ranch and feral mink

#### Kidney

Significant differences between the concentrations of chemical elements in the kidneys of ranch and feral mink were recorded only for copper, zinc and mercury (Table 2). The nephric concentrations of all these elements were lower in ranch mink.

The mean concentration of *copper* in ranch mink kidney was significantly (about 2-fold) lower compared with the mean concentration in the same tissue of all feral mink (F_4,128_ = 1.3, *P* < 0.0001). The differences were statistically significant for both sexes analyzed separately (*P* < 0.0001). The mean concentration of *zinc* in ranch mink kidney was significantly lower than the value for feral mink from all locations (F_4,128_ = 1.9, *P* = 0.002). However, differences in the mean concentrations of Zn were recorded only for ranch and feral females (*P* = 0.0006), and not for males. The mean *mercury* concentrations in kidneys of mink from the four national parks were similar. The mean concentration of mercury in ranch mink was significantly lower than the mean concentration in all feral mink (F_4,128_ = 57.32, *P* < 0.0001). Both feral males and feral females had significantly higher nephric mercury levels compared with males and females from the farm (*P* < 0.0001).

#### Liver

Similarly to kidney, significant differences were found between ranch and feral mink in the concentrations of copper, zinc and mercury in their livers. In addition, hepatic selenium, chromium and manganese concentrations differed significantly between ranch and feral mink, but only in animals from one or two national parks (Table 3).

The mean *copper* concentration in the livers of ranch mink was significantly (about 2-fold) higher than that recorded in feral mink (F_4,128_ = 3.11, *P* = 0.0001). These differences between ranch and feral mink were significant for both males (*P* = 0.0001) and females (*P* = 0.01). The mean hepatic *zinc* concentration varied significantly between ranch and feral mink (F_4,128_ = 3.57, *P* = 0.001). However, there were no significant differences in mean hepatic zinc concentration between ranch and feral mink males or females. The mean *selenium* concentration in livers of ranch mink was significantly lower than that of feral mink (F_4,128_ = 3.34, *P* = 0.002). The selenium burden differentiated ranch and feral females (*P* = 0.04), but not the males. The mean *mercury* concentration in the livers of ranch mink was significantly lower than that in feral mink (F_4,128_ = 21.72, *P* < 0.0001). Both male and female feral mink had significantly higher hepatic mercury levels compared with ranch males and females (*P* < 0.0001).

### Variation in copper, zinc and mercury concentrations in tissues of feral mink

**Fig. 2:**
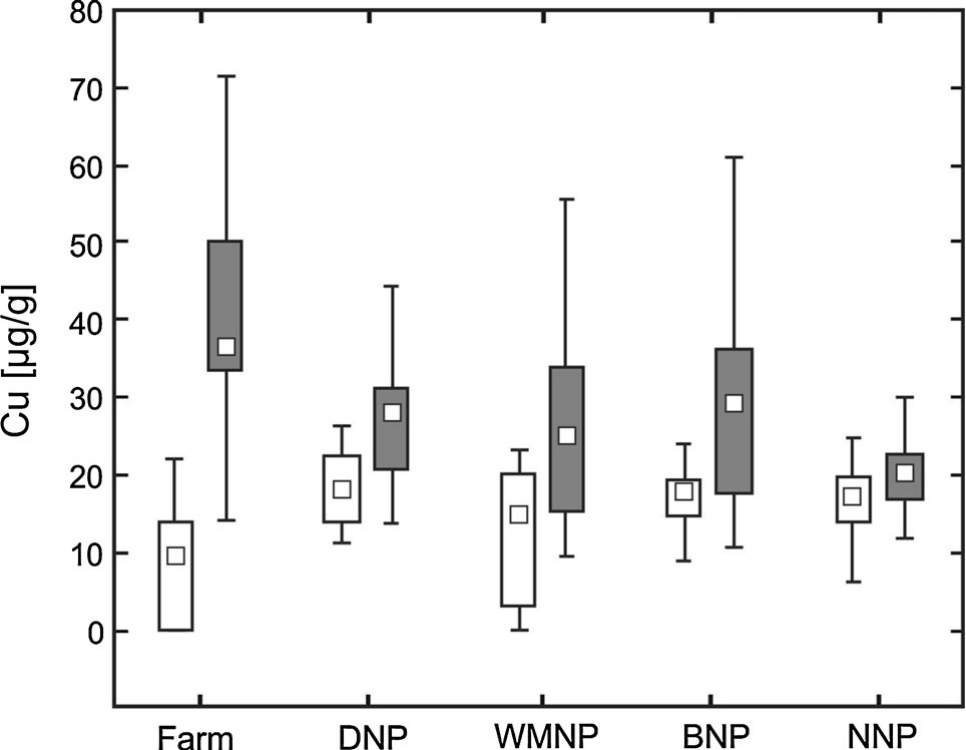
Concentrations of copper in kidney (*white box plots*) and liver (*gray box plots*) in ranch mink and feral mink (µg/g) on a dry weight basis

Mean *copper* concentrations in kidney were similar in feral mink from all national parks, although variation among individuals within each park, particularly those from WMNP, was high (Fig. 2). The mean concentration of nephric *copper* in ranch mink was significantly lower than that in feral mink from DNP (*P* = 0.0002), BNP (*P* = 0.006) and NNP (*P* = 0.008), but similar to the mean level in animals from WMNP (Fig. 2). The mean values for hepatic *copper* were similar in feral mink from the four national parks (Fig. 2) and any differences were not statistically significant. However, the mean hepatic copper concentration in mink from the national parks was significantly lower than that recorded in ranch mink (DNP *P* = 0.007; WMNP *P* = 0.007; BNP *P* = 0.006; NNP *P* < 0.0001).

The *zinc* concentration in kidney of feral mink varied between the animals in the four national parks, with the following mean values: 70.23 (22.41) μg/g in WMNP, 76.22 (15.46) μg/g in NNP, 92.92 (63.42) μg/g in DNP, and 115.40 (67.01) μg/g in BNP. There were significant regional differences in the zinc concentrations in the kidneys of mink from WMNP and BNP (*P* = 0.002), and mink from BNP and NNP (*P* = 0.014). Similar zinc levels in liver were recorded in ranch mink (71.62 ± 31.69 μg/g) and feral mink from WMNP and NNP (67.22 ± 21.99 and 82.73 ± 26.71 μg/g, respectively). Higher mean hepatic concentrations of zinc were found in mink from DNP (112.22 ± 79.50 μg/g) and in those from BNP (135.38 ± 71.13 μg/g). Significant differences in zinc concentration were found for the following pairs: DNP versus WMNP (*P* = 0.01); WMNP versus BNP (*P* = 0.0002), and BNP versus NNP (*P* = 0.002).

**Fig. 3:**
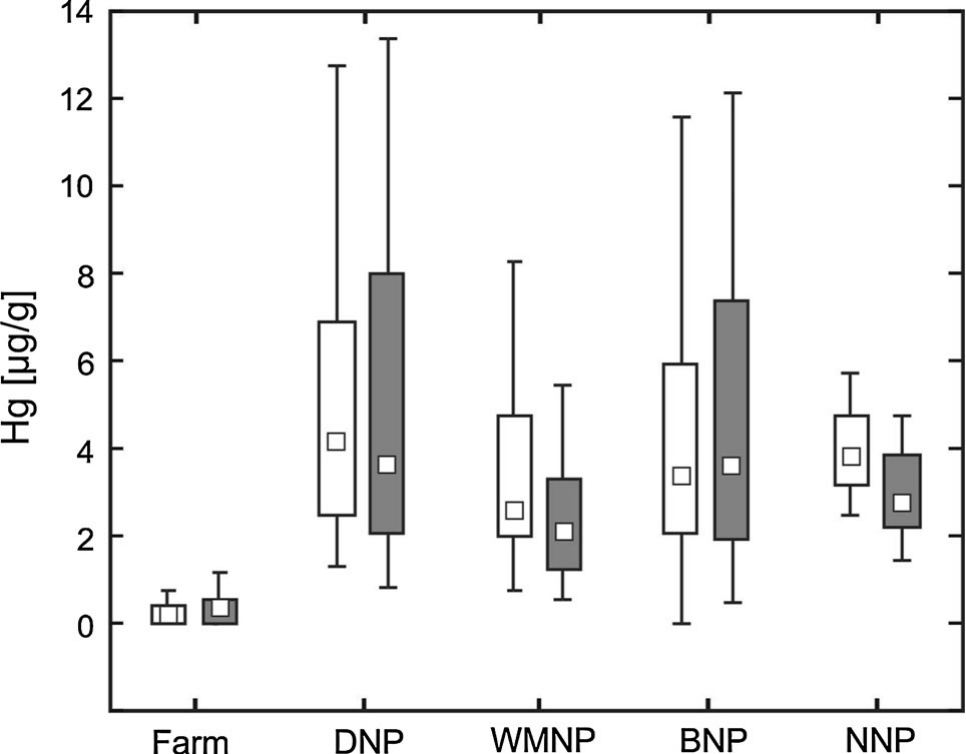
Concentrations of mercury in kidney (*white box plots*) and liver (*gray box plots*) in ranch mink and feral mink (µg/g) on a dry weight basis

Mean *mercury* concentrations in the kidneys of mink from the four national parks were similar and any differences between these feral populations were not significant (Fig. 3). However, the mean level of nephric mercury for each feral population differed significantly from that of ranch mink (*P* < 0.0001).

The mean mercury concentrations in feral mink livers also did not vary significantly between the four feral populations (Fig. 3), with one exception: hepatic mercury in mink from DNP was significantly higher than that recorded in mink from WMNP (*P* = 0.02). Similarly to the nephric mercury concentration, the mean level of this element in liver in all feral populations was significantly higher than that found in ranch mink (*P* < 0.0001).

### Mercury and copper as chemical markers

**Fig. 4:**
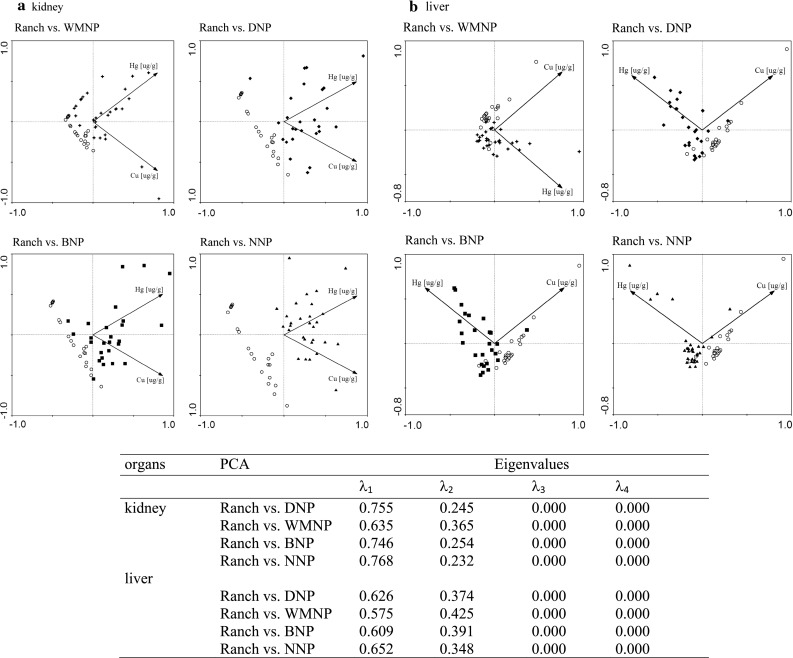
A PCA plots of nephric (**a**) and hepatic (**b**) copper and mercury concentration of ranch and feral mink from different locations. *Empty circles* denote ranch mink, other *filled symbols* denote feral mink from different national parks

Ranch and feral mink were most significantly differentiated by the kidney and liver concentrations of mercury and copper. Furthermore, the levels of these two elements in both organs were very similar in all feral populations. Plots comparing the nephric and hepatic concentrations of mercury and copper show a “spatial separation” between the ranch and feral mink; however, this separation varied according to the analyzed tissue and feral population (Fig. 4a, b).

The greatest distance separating the recorded burdens of mercury and copper in kidney was observed between ranch mink and feral mink from BNP and NNP, and the smallest distance was between the ranch mink and feral mink from WMNP (Fig. 4a). Hepatic concentrations of these two elements did not differentiate ranch and feral mink as well as nephric concentrations (Fig. 4b). The level of “spatial separation” between ranch and feral mink results from the concentrations of mercury and copper in tissues of particular mink individuals, and also from the different amount of variation in these element burdens in the compared populations. Variation in the concentration of copper among ranch mink was much higher than the variation in mercury levels.

## Discussion

Contamination in American mink, both ranch and wild-living, has been the subject of about 200 studies (Bowman et al. 2012). Most have examined the concentration of Hg and other elements such as Cd, Pb and Se (e.g. Fortin et al. 2001; Gamberg et al. 2005; Yates et al. 2005; Lake et al. 2007; Martin et al. 2011; Mayack 2012), and just a few have analyzed a broader spectrum of trace elements (Stejskal et al. 1989; Halbrook et al. 1996). The level of mercury in mink tissues is of particular concern, since this heavy metal is highly toxic and may become biomagnified in high-level predators. The effects of increased Hg concentration on neurochemical changes in mink brains have been found (Basu et al. 2005). According to analyses of Lake et al. (2007) concentrations of Hg in mink liver found in the most previous studies are high enough to state that impacts on brain neurochemistry in mink might be expected. However, the impact of mercury and other heavy metals on mink populations is not evident (Lake et al. 2007), despite the fact that some authors implicate mercury contamination as a factor that increases mortality rates and decreases reproduction rates in a population, and so may lead to population decline (Halbrook et al. 1994; Osowski et al. 1995). Wild-living mink occupy aquatic habitats that are susceptible to degradation by contaminants; therefore, despite the controversy over the value of mink as a sentinel species (Bowman and Schulte-Hostedde 2009), this carnivore is considered to be a useful indicator for assessing environmental contamination. Mink, as a top predator, are at increased risk of exposure to mercury and other chemical elements that bioaccumulate in their tissues at greater concentrations than in organisms lower in the food chain. Comparison of the mean nephric and hepatic concentrations of Zn, Pb, Cd and Cu in beavers (*Castor fiber*) (semiaquatic herbivore) from northeastern Poland (Zalewski et al. 2012) with the levels of these elements in the same tissues of mink from the same region (this study, BNP and NNP) confirms their biomagnification in predatory mink. Despite this finding, the element concentrations detected in feral mink from four national parks in Poland were low to moderate. The recorded burdens and the range of concentrations of elements such as Hg, Cu, Se, Cd and Pb were similar to those reported in previous studies of wild-living mink (e.g. Ogle et al. 1985; Poole et al. 1998; Klenavic et al. 2008; Martin et al. 2011).

Variability of element concentrations in the livers and kidneys of feral mink was higher intra-site than inter-site. Mink from a single national park often showed high variability in element concentrations, suggesting individual differences in diet composition, since the local environmental conditions were the same for all captured animals. For example, mink with a greater proportion of aquatic prey in their diet would be expected to have higher Hg concentrations in their tissues than those feeding more on terrestrial prey (Gamberg et al. 2005; Martin et al. 2011). In particular, a high proportion of fish in the mink diet may result in higher bioaccumulation of Hg. Furthermore, the concentration of mercury in top predator tissues increases proportionally to the percentage of mesopredators in their diet (Lourenço et al. 2011). Therefore, mink that feed predominantly on fish (especially predatory fish such as pike *Esox lucius* and perch *Perca fluviatilis*; see Brzeziński 2008) would be expected to accumulate higher concentrations of mercury than individuals feeding more intensively on terrestrial herbivorous prey such as small rodents (Martin et al. 2011). No dietary studies have been undertaken in the four national parks from which mink were sampled in the present study, so it is not possible to relate the contamination burdens in mink tissues to their feeding habits. In Poland, as in many other parts of the geographical range of mink, their diet varies considerably according to habitat and season; however, aquatic (fish, crayfish) and semi-aquatic prey (amphibians) usually predominate (Brzeziński and Żurowski 1992; Jędrzejewska et al. 2001; Bartoszewicz and Zalewski 2003; Brzeziński 2008).

On the large Polish farm mink are fed mainly with chicken products (75 %), and fish remains comprise only about 5 % of the fodder (Gugołek et al. 2011). The study of Stejskal et al. (1989), in which analyzed mink fodder consisted mostly of chicken (24 %) and beef (22 %), showed concentrations of mercury and several other heavy metals to be below detection limits. The differences between the diets of feral and ranch mink lead to different rates of contaminant exposure (Bowman and Schulte-Hostedde 2009; Bowman et al. 2012). Therefore, it should theoretically be possible to distinguish between these two groups of animals by the concentrations of elements they bioaccumulate as a result of the different food they consume. Our analysis confirmed this hypothesis, but only in the case of three of the 13 studied elements. The concentrations of most elements analyzed in feral mink were generally similar to those in ranch mink. Pronounced differences between feral and ranch mink were seen in the concentration of Hg, and to a lesser extent Cu and Zn. Due to the lack of significant differences in the concentrations of the other elements studied, only these three elements, and particularly mercury, may be considered as effective markers for distinguishing feral and ranch mink.

We detected significantly higher Hg burdens in the examined organs of feral mink compared with those of ranch mink. Similar but less pronounced differences were recorded in mink livers by Bowman et al. (2012). A recent study of mink in Poland by Kalisińska et al. (2012) revealed significantly higher concentrations of mercury in the kidneys and livers of feral mink (from WMNP) compared to ranch animals (90-fold and 240-fold, respectively), which is generally consistent with the results of our study. However, we detected much smaller differences between feral mink from WMNP and ranch mink in their nephric and hepatic Hg concentrations (12-fold and 4-fold, respectively). In comparison with our results, Kalisińska et al. (2012) found nephric and hepatic Hg concentrations that were lower in ranch mink and higher in feral mink, but their study was based on a very small sample (13 individuals). In our study, greater mercury concentrations were generally evident in the tissues of feral mink compared to ranch animals, but some feral individuals were characterized by very low Hg levels in kidney and/or liver, that were comparable with levels detected in mink from the farm. Four individuals from WMNP exhibited mercury concentrations that were within the range for ranch mink and much below the mean level of Hg in feral mink from this national park. Very low concentrations of Hg in feral mink tissues may suggest that such individuals are recently escaped ranch mink (Kalisińska et al. 2012). However, an alternative explanation is that these animals are feral mink that have not bioaccumulated higher Hg concentrations for other reasons, such as the predominance of terrestrial food in their diets. Feral mink individuals may specialize in hunting particular prey (Sidorovich 2000; Brzeziński 2008), so the observed variation in the levels of pollutants should be higher compared to ranch mink. Indeed, we recorded much higher variation in the level of Hg in feral mink tissues. However, the larger variation of Hg concentration in feral mink from WMNP compared with those from other national parks is probably related to the higher number of farm escapees at this site (Zalewski et al. 2010).

The recorded differences in Hg contamination levels in feral mink and ranch mink escapees result from their different diets in the wild and on farms. Bowman et al. (2012) recently suggested that such differences in contamination burden may be conserved in farm escapees and hybrids due to their different behaviour and physiology compared to wild mink. However, it has yet to be proven that mink which have escaped from farms have feeding habits that are different to those of wild-living mink. Without such feeding differences and due to the rapid bioaccumulation of Hg, the mercury burdens in mink tissues will not permit farm escapees that have survived in the wild for a long time to be distinguished from individuals born in the wild. Our results indicate that the Hg concentration in kidney more precisely differentiates feral and ranch mink (or ranch escapees) than the Hg concentration in liver. The concentration of Hg was similar in both organs, however, other authors have recorded higher concentrations of mercury in liver than in kidney (Fortin et al. 2001; Gamberg et al. 2005).

High Cu concentrations in ranch mink livers result from the high concentration of this element in feed additives in the mink fodder. The process of normal hair and wool pigmentation requires copper. Copper is a cofactor of the enzyme polyphenyl oxidase, which catalyzes the conversion of tyrosine to melanin, it is required for the incorporation of disulfide groups into keratin in wool and hair, and the liver is the main storage organ for this element (Church and Pond 1989; Stejskal et al. 1989).

Besides the mercury burden, the mean concentration of Cu was the most significant means of distinguishing the two groups of mink: compared with feral animals, we found lower concentrations of Cu in ranch mink kidney and higher levels in liver. The Cu concentration was higher in the liver than kidney, as has been observed in previous studies (Stejskal et al. 1989). Higher concentrations of Cu (and Zn) in mink liver compared to kidney were also reported by Halbrook et al. (1996). The concentrations of heavy metals in various tissues are often highly correlated (Klenavic et al. 2008). If mean Hg and Cu concentrations are significantly different in ranch and feral mink, one might expect positive (for kidney) or negative (for liver) correlation of the levels of these elements in individual mink tissues; however, such correlations have not been identified.

We found that the concentrations of most elements did not differ between the sexes, and the lack of a sex effect has been recorded in several previous studies on mink contamination (Hg—Lake et al. 2007; Klenavic et al. 2008; Hg, Se, Cd, Pb—Martin et al. 2011). However, such differences have been recorded in the case of mercury in some studies: concentrations of Hg were greater in female than male mink tissues (Gamberg et al. 2005) or just in livers (Yates et al. 2005). Mayack (2012) recorded higher concentrations of Hg and Cd in female mink and explained this finding by gender differences in relative growth. On the other hand, it was found that lead concentrations were higher in male mink (Mayack 2012). The feeding habits of mink are also gender related (Birks and Dunstone 1985), and result mostly from the well pronounced sex-related size dimorphism. Body mass of mink individuals may affect the selection of prey, even within one gender (Zalewski and Bartoszewicz 2012), and should therefore be considered an important parameter in explaining the variation in contamination in wild mink. In the present study, we detected correlations between body size and the concentrations of chromium, zinc and manganese, but the nature of these relationships varied between the two groups of mink (feral and ranch) and between sexes.

The bioaccumulation of contaminants is usually correlated with the age of carnivores (including mink), and is higher in older individuals (Hyvärinen et al. 2003; Martin et al. 2011). However, some studies have failed to detect a relationship between age and the amount of heavy metals in the tissues (Mierle et al. 2000; Ben-David et al. 2001; Gamberg et al. 2005; Lake et al. 2007; Klenavic et al. 2008). In the present study, we also did not find any correlation between the concentration of the majority of elements and mink age. This lack of such a relationship may be related to the examination of a narrow age range (Yates et al. 2005), and the small number of mink older than 2 years that were analyzed by us limited the representation of animals that could bioaccumulate elements for a long time. Therefore, dividing mink into two age groups reduces the opportunity to detect age-related concentrations of chemical elements, which may be more likely in long-lived animals. This fact is related to the high mortality rate of feral mink, whereby individuals of over 1 year old comprise only a small fraction of the population. Furthermore, the initial mercury concentrations in mink may be high, possibly due to maternal transfer, and the limited relative increases in Hg with age suggests that the hepatic mercury burden is, to a large extent, established by Hg exposure during early development (Mayack 2012).

## Conclusions

Evaluation of the concentrations of some trace elements in mink tissues may not only indicate environmental contamination, may be used as a means of estimating the inflow of farm escapees to feral populations. As suggested recently by Bowman et al. (2012), the identification of quantitative criteria that allow ranch and wild-living mink to be distinguished, is highly desirable. The results of our study are not sufficient to unequivocally establish such criteria, but they suggest that Hg and probably also Cu should be considered as indicators of the origin of individuals living in feral populations. Furthermore, comparison of the proportions of mercury and copper in mink tissues may serve as an index to estimate the time that escapees have been living in the wild. After escape, the concentration of Hg should increase and that of Cu should decrease. The analysis of Hg and Cu burdens in mink tissues could support estimates of the proportion of farm escapees based on genetic or stable isotope analyses. Further research to test the efficacy of combining these methods, and to determine the mercury and copper burdens in mink prey and fodder, is required to identify more precise indicators of individual mink life histories and methods for assessing ranch mink inflow to feral populations.
